# From Biologically Active Complexes with Camphor-Derived Ligands to Biologically Active Biomaterials

**DOI:** 10.3390/antibiotics15080725

**Published:** 2026-07-25

**Authors:** M. Fernanda N. N. Carvalho, Joana P. Costa

**Affiliations:** Centro de Química Estrutural, Institute of Molecular Sciences and Departamento de Engenharia Química, Instituto Superior Técnico, Universidade de Lisboa, 1049-001 Lisboa, Portugal

**Keywords:** camphor coordination compounds, antimicrobials, anticancer agents, silver, functionalization of biomaterials

## Abstract

The aim of this mini-review is to highlight the significant medicinal potential of camphor coordination compounds as anticancer and antimicrobial agents. The appropriate selection of the metal center and rational design of the camphor derivatives can optimize the biological properties of these compounds, leading to the development of molecules with combined anticancer and antimicrobial activities. A promising area for future research is the incorporation of biologically active camphor coordination compounds into biocompatible materials (e.g., hydroxyapatite or hydrogels) to develop functional composites and wound dressings with enhanced therapeutic properties.

## 1. Synopsis

Scientific progress has been driven by ideas that generate hypotheses which—through trial, error, improvement, and some luck—lead to success. New chemical and biotechnological processes and industries represent the success of ancient and modern discoveries that changed not only the human style of life but also its quality and longevity. However, not just benefits exist! Risks are inherent to evolution because it is not possible to foresee and prevent all disadvantages of novel products and technological processes.

We are currently moving from the Fourth Industrial Revolution [1], during which traditional trial-and-error approaches have evolved into highly specialized, data-driven solutions, towards the Fifth Industrial Revolution, where humans and machines are expected to interact and collaborate [2]. Such transformations will profoundly shape our world and the future of new generations, posing a variety of challenges and risks to human life and the environment. In the context of human life, increasing longevity and stress will require much attention. In the context of sustainable development, the benefits of technological advances require solutions to global warming, climate change, and plastic pollution to less perceptible risks such as debris in interplanetary space—but not just those. Health care therapies also require new solutions to face new maladies and acquired resistance of microorganisms and cells to existing medicines.

Unlike cancer, which became a major global health concern during the 20th century, infectious diseases have been recognized and fought since ancient times. Infection treatment historically relied on natural products that demonstrated efficacy through empirical trial and error, while consistency and reproducibility were fundamental criteria for their therapeutic application.

The discovery of penicillin initially appeared to have resolved the problem of infections. However, the misuse and abuse of antibiotics led to microbial adaptation and the emergence of resistance, reviving global concerns about infectious diseases and prompting a renewed interest in traditional therapies while also intensifying efforts to discover new synthetic compounds and innovative treatment strategies. Coordination compounds, which combine metals with organic ligands, may represent promising alternatives to conventional organic antimicrobials. Furthermore, metals other than platinum may exhibit fewer adverse effects than cisplatin in cancer treatment.

## 2. Camphor: From an Old Medicine to Much More

Camphor, obtained from the wood of the camphor laurel (*Cinnamomum camphora* L.), is one of the oldest natural products with a wide range of applications. It was used by the Egyptians (as early as 3400 BC) for its antimicrobial and fragrant properties and as a preservative to prevent the decay of mummies. Other cultures, such as the Chinese, recognized camphor’s beneficial effects in treating respiratory and muscular ailments [3]. More recently (the 14th century), camphor was also used as a fumigant during the Black Death [4]. Camphor herbal infusion’s aphrodisiac properties are appreciated by men in some African countries. However, their abuse involves considerable risk due to camphor oral toxicity [5].

The antimicrobial properties of camphor used in traditional medicine are now supported by scientific evidence [4,6]. At present, camphor-containing products are commercially available in pharmacies for the treatment of cough, pain, and itching. As an analgesic, the antinociceptive properties were associated with the desensitization of the transient receptor potential vanilloid 1 (TRPV1) protein and the blocking of the ion channel (TRPA1), that act as sensors for pain detection [7]. Potential anticancer properties have recently emerged for several camphor derivatives [8].

Non-medicinal uses of camphor include, for example, insect repellents [9] or additives for food-packaging films [10].

The biological properties of camphor are driven by its chemical characteristics, which are defined by the bicyclic structural arrangement of its skeleton, imparting rigidity to the molecule while maintaining a degree of structural flexibility. Additional groups enable interactions with an extended range of biological entities [4,11]. The chiral character of the camphor molecule tunes the interaction with DNA and other specific receptors [12], thereby contributing to its biological activity.

Over time, studies on expanding [13] or breaking [14,15] one of the bicyclic units of camphor, or the replacement of H or O atoms by substituent groups, have enabled the synthesis of numerous camphor derivatives, [16,17,18,19,20,21,22,23,24,25,26,27,28,29,30,31,32,33] broadening its prospective applications beyond the traditional biological ones (e.g., Alzheimer’s disease [34], catalysis [35,36,37,38,39,40], or asymmetric synthesis [41,42,43,44]).

Among the recently explored applications of camphor derivatives, the potential activity against microorganisms other than bacteria, such as viruses (e.g., influenza, [45,46,47] vaccinia, Marburg [48], Ebola [49]) or parasites (e.g., *Leishmania* [50], *Toxocara canis* [51]), has emerged. Additionally, camphor derivatives as optically active compounds [52] have proven to be suitable visible-light photo initiators for in situ hydrogel photopolymerization [53], promising materials for solar thermal energy storage systems [54] that act as molecular motors driven by light or heat [55].

As ligands, camphor derivatives promote the formation of a broad range of coordination compounds with tunable structural and electronic properties, thereby expanding their potential for multiple applications [48,56,57,58].

**Figure 1:**
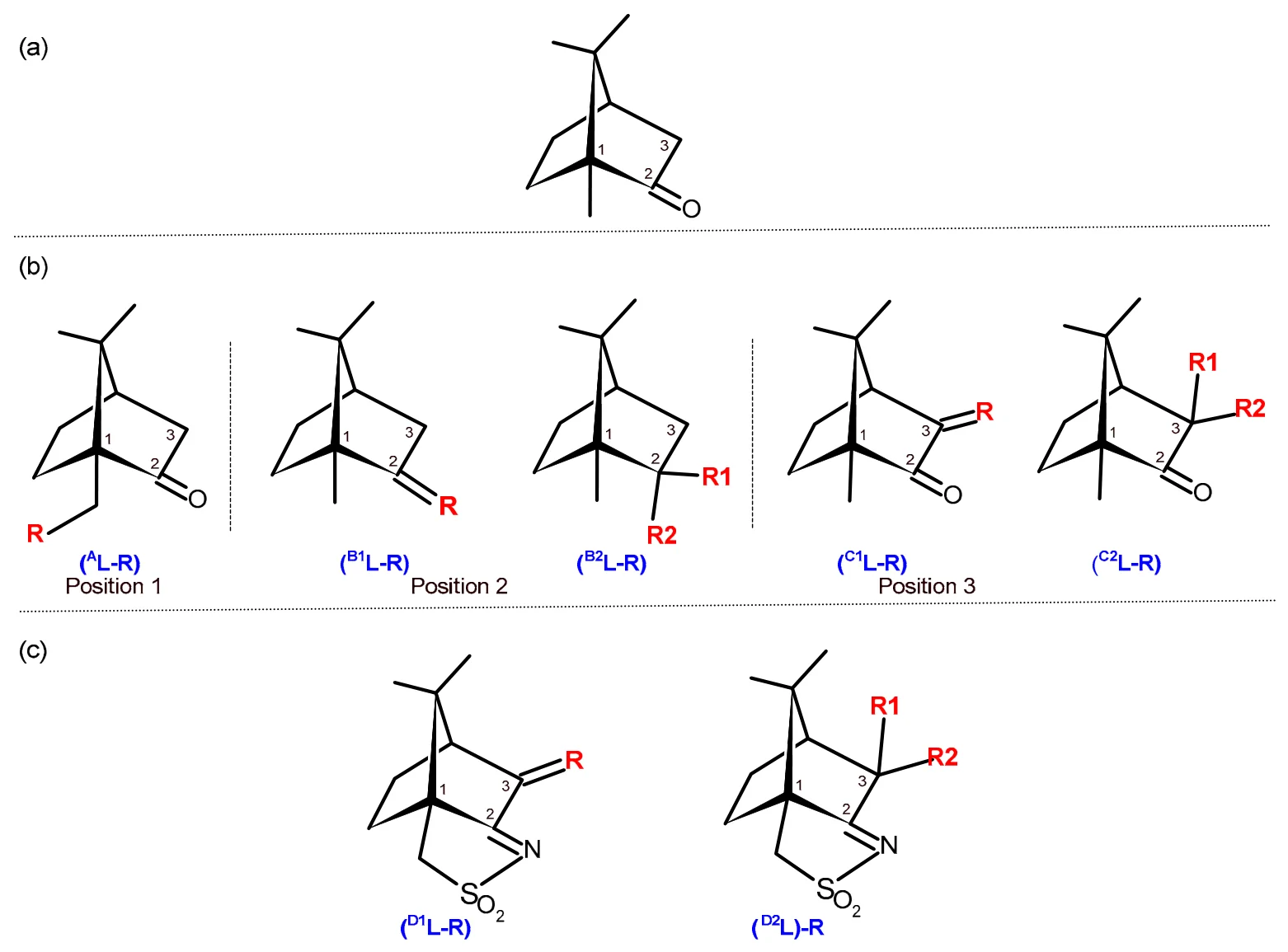
Schematic representation of camphor (**a**) and camphor derivatives with substituents at: (**b**) position 1, (^A^L-R), position 2, (^B1^L-R; ^B2^L-R) or position 3 (^C1^L-R; ^C2^L-R). (**c**) camphor sulfonic acid derivatives (^D1^L-R; ^D2^L-R).

Camphor (C_10_H_16_O, Figure 1a) by itself is a poor ligand, since its coordination to metals often involves the ketone group (>C=O), which is hindered by the bulky rigid bicyclic skeleton. Therefore, camphor derivatives capable of coordinating metal centers typically have substituents with electron-donor atoms at positions 1, 2, or 3 of camphor (Figure 1b,c) that facilitate binding to the metal. For simplicity, no camphor derivatives substituted at more than one position are displayed. Innovative methods for functionalizing the camphor scaffold through C–H activation have emerged, offering new perspectives to metal coordination [59].

The special appeal of coordination compounds in medicinal applications relies on the combination of organic and inorganic moieties, which enable reactivities distinct from organic precursors and may help to overcome microbial and/or cancer cell resistance through mechanisms different from those of the conventional drugs.

## 3. Coordination Compounds for Therapeutical Uses

The discovery of the therapeutic properties of cisplatin boosted the search for new anticancer coordination compounds. Other platinum-based drugs, such as carboplatin or oxaliplatin, were developed and adopted for therapeutic uses, while complexes such as picoplatin or satraplatin (IV) failed to meet Phase III clinical trial requests for FDA approval. Although platin looks a suitable metal to fight cancer cells, the undesirable side effects of cisplatin led to the search for coordination compounds based on more biocompatible metals. Complexes based on Ru, Ag, Au, or Ir are potential alternatives to cisplatin, aiming at lower systemic toxicity and fewer adverse side effects [60,61]. Like cisplatin, most of the complexes (Pt, Pd, Ru) with anticancer potential act through binding to DNA [62]. Alternative action modes [63] may act against cancer cells’ resistance. Several Ru complexes were identified as especially promising in reducing cancer metastasis and resisting cisplatin [64,65].

**Figure 2:**
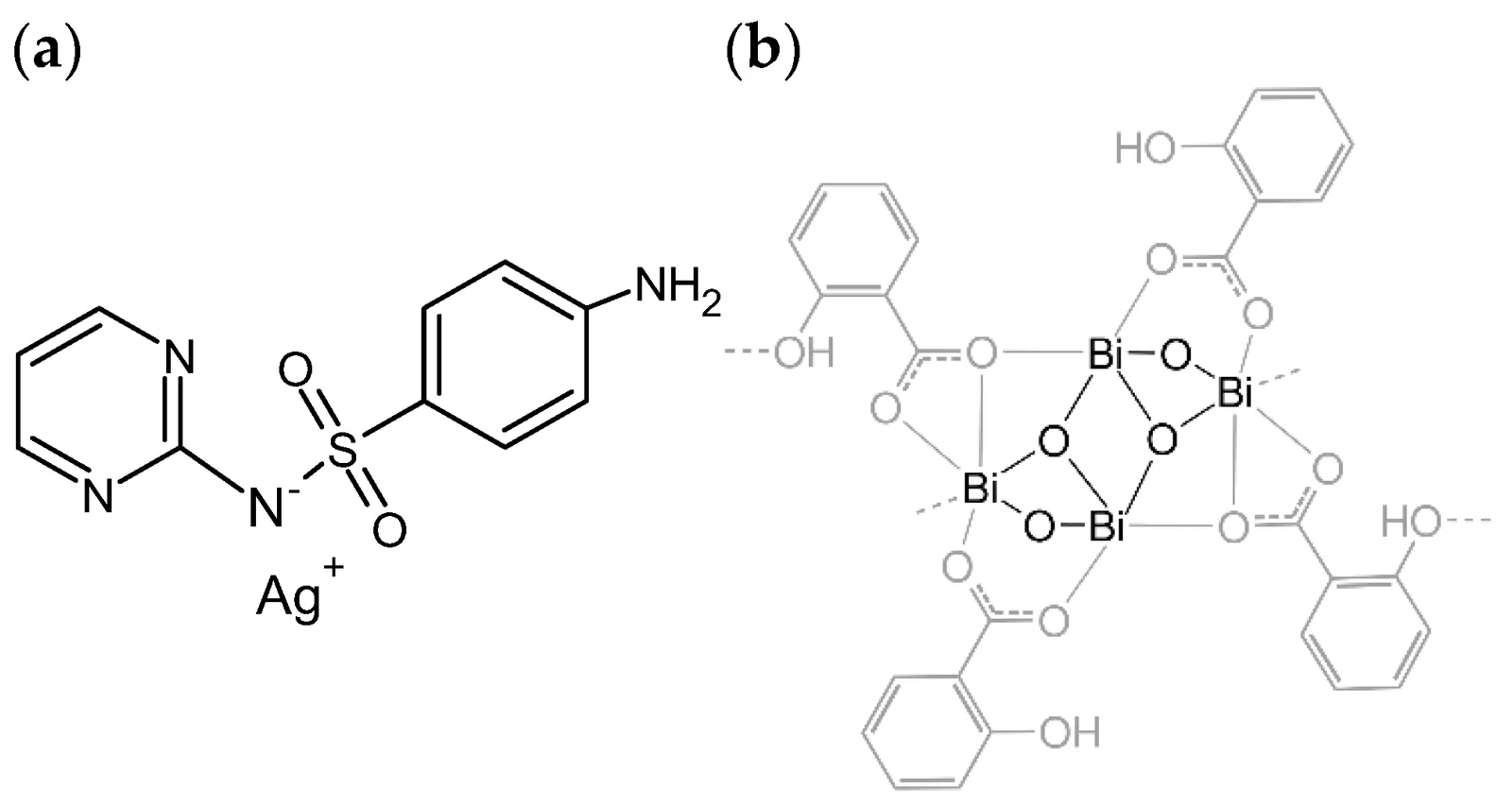
Structure of (**a**) silver sulfadiazine; (**b**) bismuth subsalicylate.

The effectiveness of metal compounds’ ability to fight cancer is recognized and accepted. However, their use as antimicrobials is less explored. A few compounds (e.g., silver sulfadiazine, Figure 2a, or bismuth subsalicylate, Figure 2b) are used as antimicrobials. However, a recent screening of the antimicrobial activity of more than 900 complexes (Pt, Pd, Ag, Ir, Ru, etc.) [66] clearly highlighted the potential of coordination compounds as alternative antibiotics towards bacteria and fungi, since they can act through mechanisms different from organic compounds (e.g., ligand exchange or release, ROS and toxic species generation, or depletion of essential substrates).

The intrinsic antimicrobial properties of camphor support the search for new compounds that combine camphor derivatives with metals to activate mechanisms of action distinct from those of currently available medicines. Metal’s characteristics vary considerably with the electronic configuration and the atom’s dimension, according to their positions in the periodic table. Therefore, the properties of the camphor complexes vary considerably by changing the metal, even if the ligand is the same.

### 3.1. Bioactive Camphor-Derived Complexes

While preserving the bicyclic framework of the camphor molecule, numerous camphor derivatives were designed, and in many cases synthesized, resulting in a library of camphor-based compounds with a wide scope of properties and potential applications [67,68].

This mini-review focuses on the antimicrobial and anticancer activity of complexes with camphor-derived ligands with substituents at positions 1, 2, or 3 (Figure 1b) or camphor sulfonic acid derivatives with substituents at position 3 (Figure 1c). Three binding modes will be considered: (i) coordination through the atom directly attached to the camphor skeleton; (ii) cooperative coordination of one atom directly attached to the camphor skeleton and another at the camphor substituent; and (iii) coordination through one or more atoms of the substituent (R), but not the atom directly bound to the camphor framework.

The properties of the camphor-derived coordination compounds are largely determined by the nature of the metal center. The metal’s electronic and redox characteristics are critical in determining complex properties, despite the steric and electronic effects of the ligands fine tuning their reactivity.

Silver is one of the metals that most effectively imparts antimicrobial properties to coordination compounds [69,70] with the concomitant ability to enable anticancer properties.

In this mini-review, the values of the compound’s biological activity are denoted as Minimum Inhibitory Concentration (MIC) and Inhibitory Concentration for 50% of the cells (IC_50_), respectively, for antimicrobial and cytotoxic activities.

#### 3.1.1. Complexes with Ligands Substituted at Position 1 (^A^L-R)

There is a wide library of camphor derivatives with substituents at position 1 (^A^L-R, Figure 1), some of which were assessed for antimicrobial properties [12,20,26,30,33]. However, not many coordination compounds with such types of ligands were synthesized, and even less were assessed for biological activity. The silver camphorsulfonato complexes [Ag(camphSO_3_)(PR_3_)] evidence excellent antifungal activity against *C. albicans* and *C. neoformans* (1–16 μg/mL) and moderate to high antibacterial activity (4–32 μg/mL) towards *S. aureus*, *E. coli*, *K. pneumoniae*, *P. aeruginosa*, and *A. baumannii* [71], showing their potency as antimicrobials.

#### 3.1.2. Complexes with Ligands Substituted at Position 2 (^B^L-R)

The substituents at position 2 can bind to the camphor skeleton through a double (^B1^L-R) or single (^B2^L-R) bond (Figure 1), consequently promoting distinct interactions with metals and complex properties.

##### Biological Activity

Most of the biologically active coordination compounds have camphor ligands of type ^B1^L-R, with the R substituent binding to the metal by nitrogen, sulfur, or carbon atoms. Coordination through the nitrogen atom of imine substituents at position 2 (Figure 1) often has the cooperative coordination of hetero- or homoatoms of the substituent moiety (R), forming chelate-type complexes [72]. That is the case of camphor dinitrophenyl hydrazine complexes [Mn(^B1^L-R)(H_2_O)_3_]·H_2_O and [Fe(^B1^L-R)(H_2_O)_3_]·H_2_O (R=C_6_H_3_(NO_2_)_2_NHN), which behave as moderately active against bacteria (*S. aureus*, *E. coli*, and *Salmonella typhi*) and fungi (*Aspergillus flarus*, *Mucor iudicus*, *Asphergillus niger*) [73].

**Figure 3:**
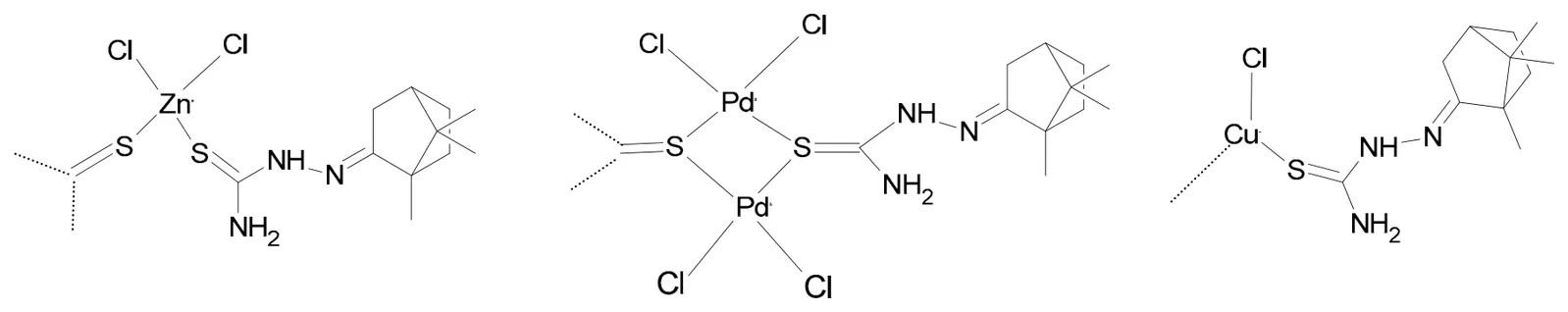
Thiosemicarbazone camphor complexes.

A different situation is observed for ligands of the ^B1^L-R type, which coordinate the metal through donor atoms other than the one directly attached to the camphor skeleton. This behavior is exemplified by the thiosemicarbazone camphor complexes [Zn(^B1^L-R)_2_Cl_2_], [Pd_2_(^B1^L-R)_2_Cl_4_], and [Cu(^B1^L-R)Cl], in which the ligand coordinates the metal through the sulfur atom (Figure 3). These complexes are active against MCF-7 breast cancer cells, with IC_50_ values in the range of 9.8–14.0 μM. The Cu(I) complexes display the lowest IC_50_ value [74].

#### 3.1.3. Complexes with Ligands Substituted at Position 3 (^C^L-R)

As mentioned above for camphor derivatives substituted at position 2, two possibilities exist for substituents at position 3 (^C1^L-R, ^C2^L-R) (Figure 1). However, most of the biologically active complexes have ^C1^L-R type ligands.

**Figure 4:**
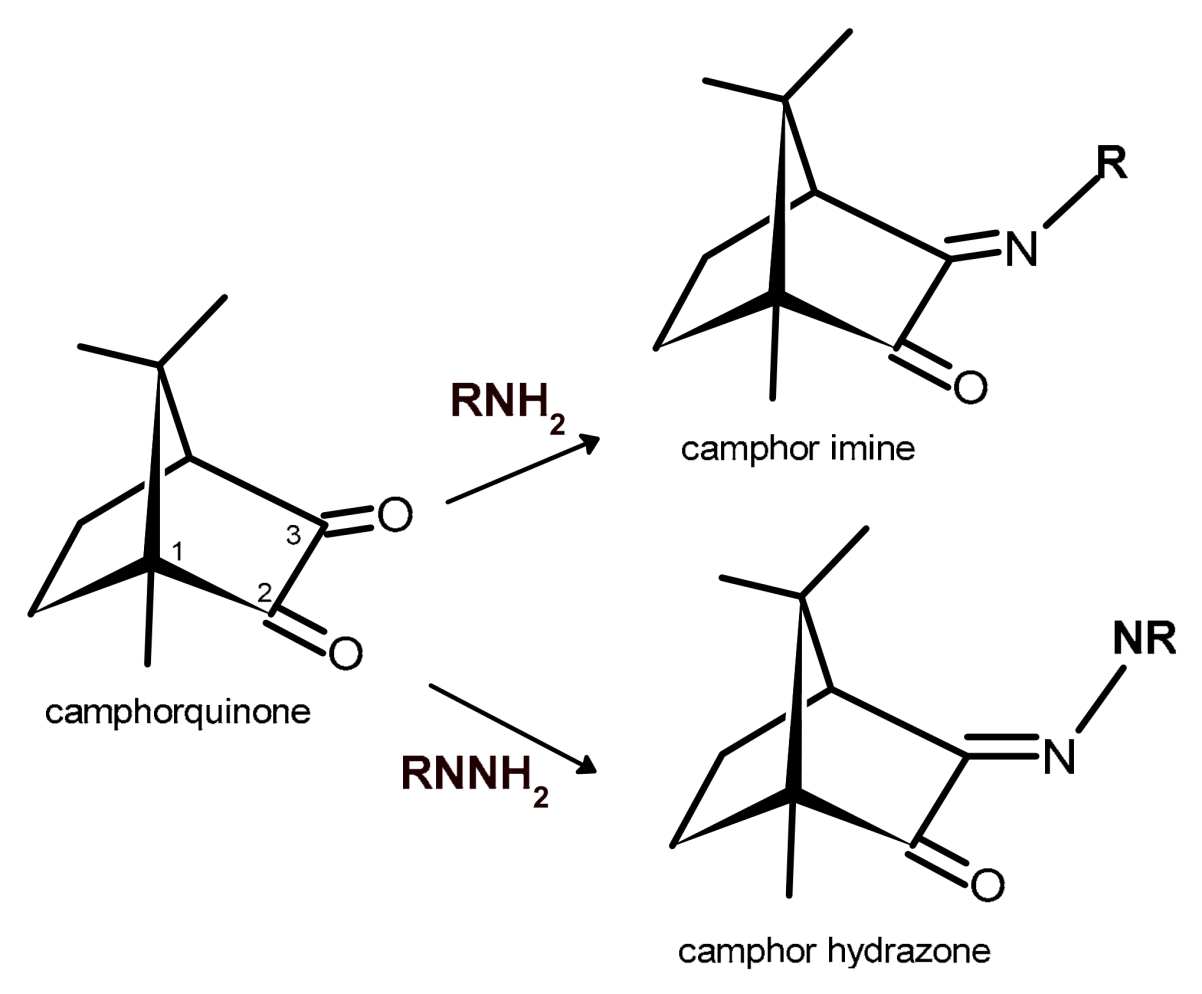
Camphor imine/hydrazone formation from camphorquinone.

Camphorquinone, obtained from the oxidation of camphor (e.g., by selenium dioxide) [75], is the precursor of a wide variety of camphor ^C1^L-R derivatives. Condensation of primary amines (RNH_2_) or hydrazines (RNNH_2_) with the ketone group (Figure 4) yields imine-type ligands (^C1^L-R) that typically coordinate metals through the imine nitrogen atom. Camphor hydrazones (i.e., RNNH_2_ derivatives) can coordinate through any of the nitrogen atoms. In some cases, cooperative stabilization through the camphor ketone oxygen atom also occurs [76].

**Figure 5:**
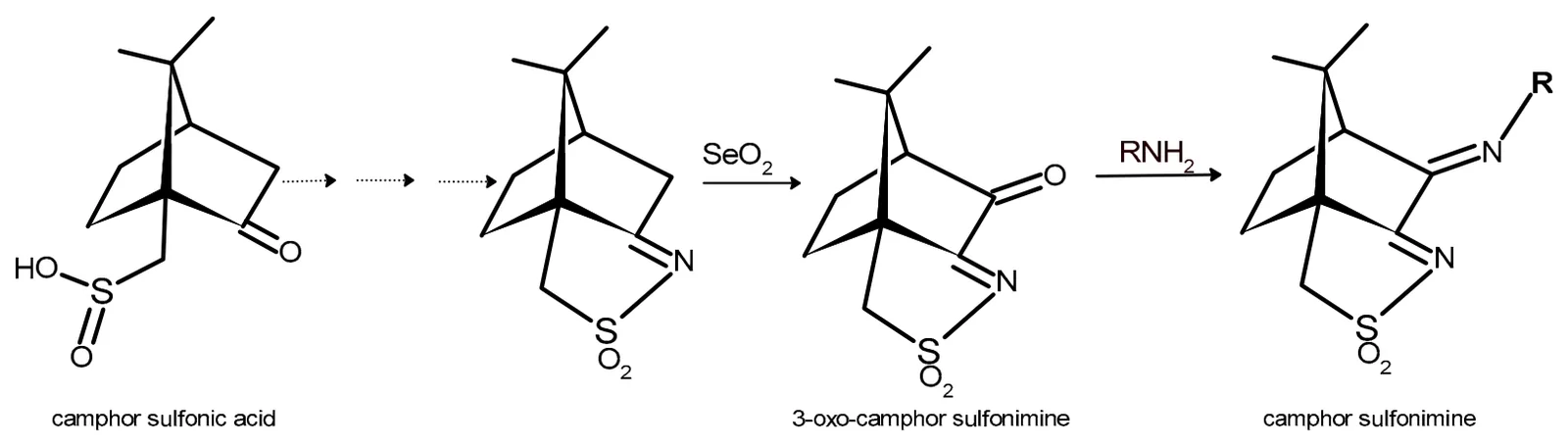
Sequential synthesis of camphor sulfonimine from camphor sulfonic acid.

Camphor sulfonic acid (Figure 5) prompts the formation of imine-type derivatives that allow structural, electronic, and coordinative comparison to camphor derivatives. Camphor imine (^C1^L-R) and camphor sulphonic imine (^D1^L-R) compounds have in common the bicyclic skeleton of camphor. A schematic route to obtain ^D1^L-R derivatives from commercially available camphor sulfonic acid through 3-oxo-camphorsulphonime is displayed (Figure 5).

A considerable number of camphor imine and camphor hydrazine complexes with ^C1^L-R or ^D1^L-R were successfully synthesized and assessed for antimicrobial and/or anticancer activity.

##### Biological Activity

As mentioned above, the metal center largely determines the properties of the complex; silver is particularly well suited to promote antimicrobial activity, whereas platinum and gold are more effective as cytotoxic agents.

**Figure 6:**
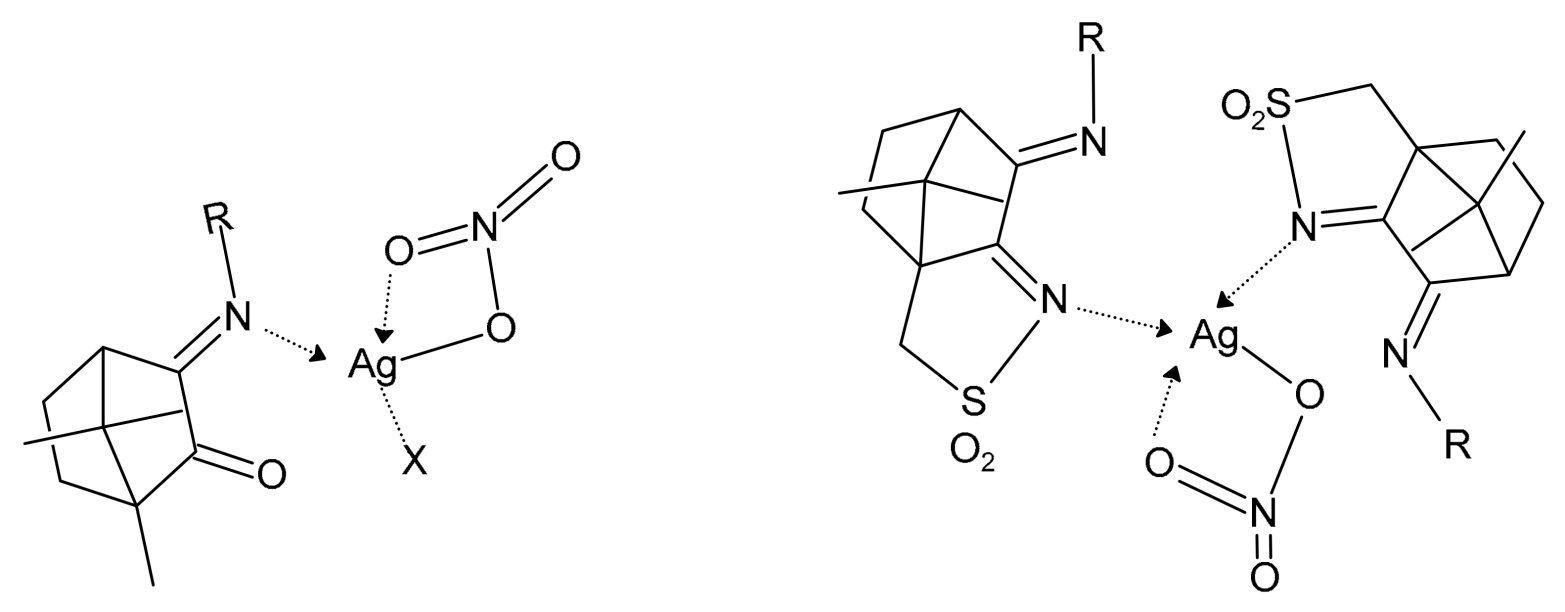
Exemplificative structural arrangement of Ag(I) complexes with ^C1^L-R (**right**) and ^D1^L-R (**left**) ligands.

Several silver nitrate complexes {Ag(NO_3_)(^C1^L-R)_n_} (n = 1,2) and {Ag(NO_3_)(^D1^L-R)_2_} (Figure 6) with camphor imine ligands (substituted at position 3) exhibit antibacterial and/or antifungal activity.are not needed.

Complexes {Ag(NO_3_)(^C1^L-R)_n_} (n = 1,2) show particularly high activity (low MIC values) towards *Candida* strains such as *C. glabrata* (15.6–62.5 μg/mL), *C. tropicalis* (3.9–31.3 μg/mL), and *C. parapsilosis* (2–15.6 μg/mL) but fail to fight *Candida albicans* [77]. However, keeping the ligand but replacing the nitrate co-ligand by hydroxide or oxide confers to the new complexes [Ag(OH)(L)_2_] (L = ^C1^L-R or ^D1^L-R) [(Ag^C1^L-R)_2_(μ-O)] (L = ^C1^L-R or ^D1^L-R) activity against *C. albicans* (7.8–31.3 μg/mL), emphasizing the role of the characteristics of the metal core in the compound’s properties [78]. In addition to antifungal activity, the {Ag(NO_3_)(^C1^L-R)} and {Ag(OH)(^C1^L-R} complexes also displayed antibacterial activity towards Gram-negative *E. coli* (ATCC25922), *P. aeruginosa* (477), and *B. contaminans* (IST408) and Gram-positive *S. aureus* Newman.

The {Ag(OH)} complexes generally exhibited lower MIC values than the corresponding {Ag(NO_3_)} complexes with analogous ^C1^L-R ligands. In some cases, the values differ by one order of magnitude, highlighting the superior antibacterial activity of the hydroxy silver camphorimine complexes (Table 1). Regarding the activity of nitrate complexes with ^C1^L-R- or ^D1^L-R-type ligands, the MIC values are slightly lower for the ^D1^L-R-based complexes (Table 2), consistent with their higher antibacterial activity. All complexes (Table 1 and Table 2) exhibit greater antibacterial activity against Gram-negative than against Gram-positive bacterial strains [79]. Based on the published results, the discussion on structure–activity relationships seems speculative because of the limited dataset.

Some of the camphor imine complexes [Ag(NO_3_)(^C1^L–R)_n_] (n = 1,2); [Ag(NO_3_)(^D1^L–R)_2_] (Figure 6) combine antimicrobial and cytotoxic activity towards different types of cancer cells [81], with selectivity (SI = IC_50HDF cell_/IC_50cancer cell_) depending on the imine substituent (Table 3). The aromatic nature of the R substituent influences the lipophilicity of the complexes and apparently plays a greater role in determining the selectivity than the anticancer activity. This is supported by the similar IC_50_ values observed against the A2780 or HOS (ATCC, CRL-1543) cell lines displayed by the {AgNO_3_} complexes (Table 3), as well as complexes containing other metal cores (e.g., [Ag(^C1^L-R)_2_(µ-O)] (R = 4-CH_3_(C_6_H_4_)) (8.2 ± 4.9 μM); [Ag(OH)(^D1^L-R)]·CH_3_COOH (R = 4-NH_2_(C_6_H_4_)) (6.1± 2.2 μM, SI = 7.5), regardless of whether the R substituent is aromatic or aliphatic. In contrast, the selectivity indices (SI) varied considerably among these compounds. SI is a significant parameter when considering potential pharmacologic applications.

**Figure 7:**
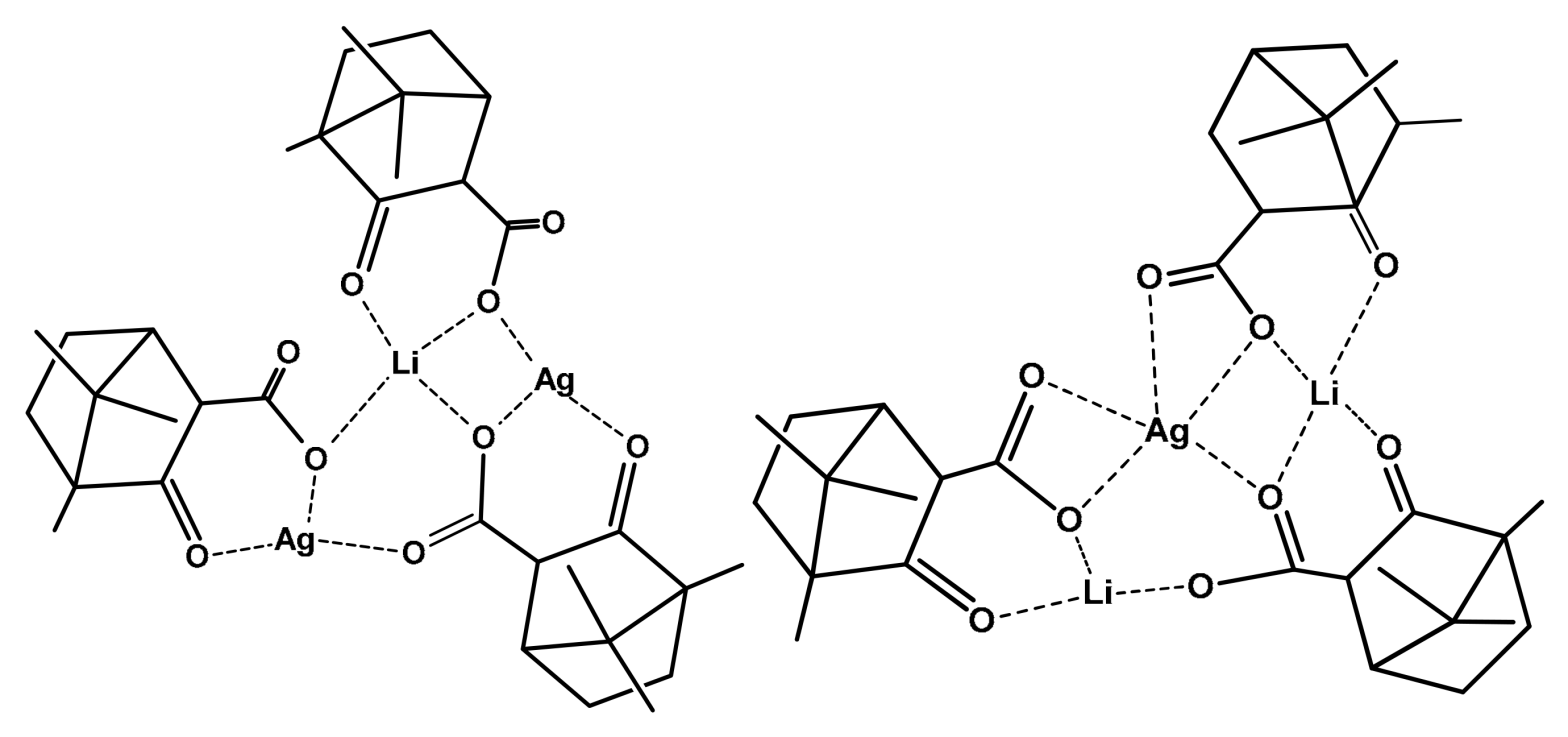
Structural arrangement of Ag(I) camphor carboxylate complexes.

In addition to camphor imine Ag(I) complexes (Figure 7), several silver camphor carboxylate and carboxamide complexes showed cytotoxic activity. Their activity against ovarian cell lines A2780 (IC_50_, 3.8–7.3μM) and platinum-resistant A2780cisR (IC_50_,11–14 μM) was reported to be considerably higher than that of cisplatin (20.7 ± 5.6 μM and 35.7 ± 11 μM, respectively), which is the drug in clinical use. Of note is the toxicity of the Ag(I) carboxylate complexes towards non-tumoral cell line HEK 293 (62.2 ± 16 μM) is low according to a considerable selectivity (16 > SI > 8.5) [84].

Although less numerous than silver, some copper- and iron-based camphor complexes were reported to have anticancer properties. Cu(I) complexes [(CuX)_2_(^C1^L-R)]_n_ (polymer) and [{(Cu(^C1^L-R)}_2_(^C1^L-R -X)_2_] (dimer) revealed moderate activities towards colon HT-29 [85] and ovarian A2780 [86] cancer cells. In contrast, the Cu(II)-related complexes [CuX_2_(^C1^L-R)] displayed no activity, highlighting the relevance of the metal oxidation state (Cu(I) or Cu(II)) for the process.

**Figure 8:**
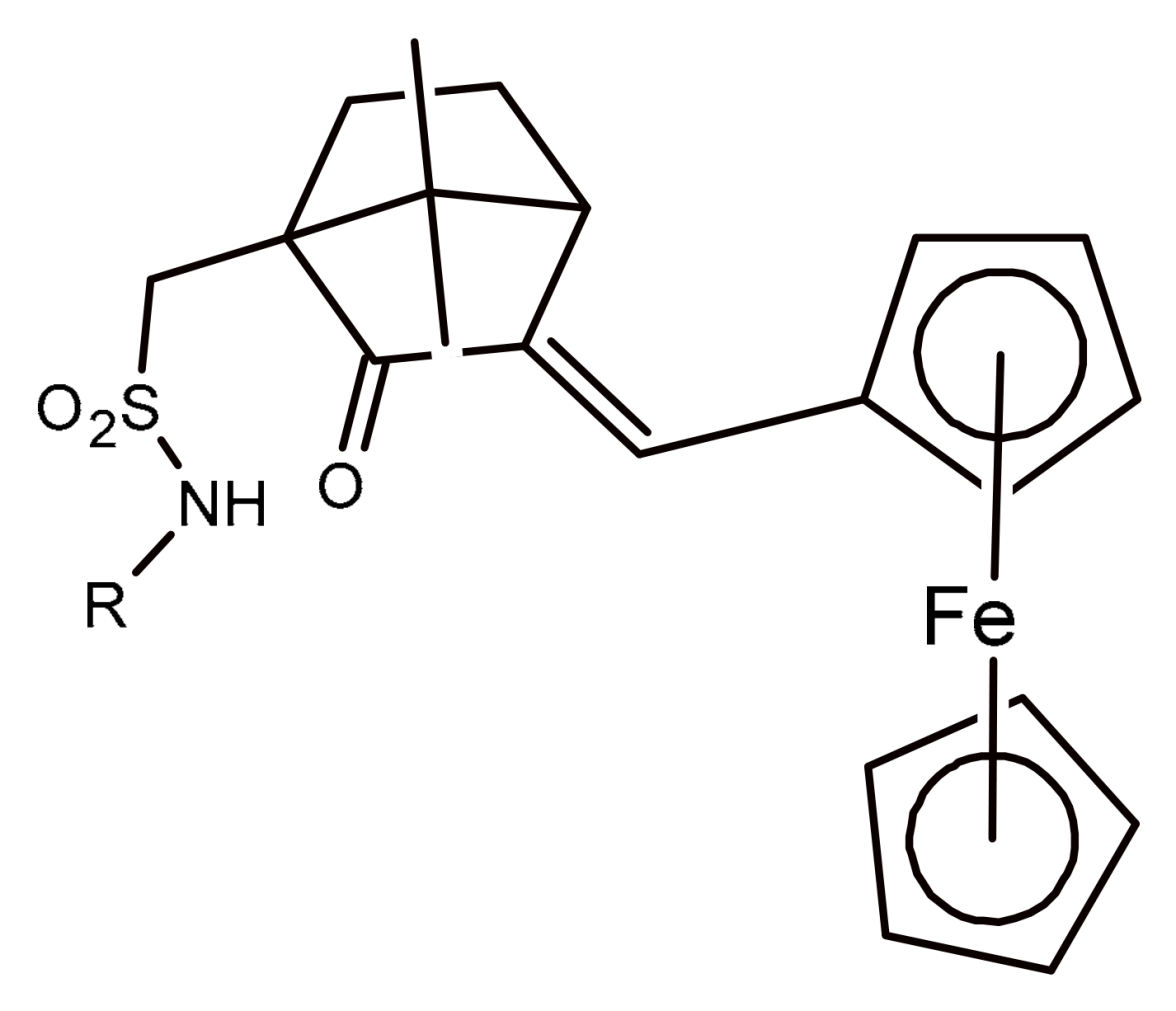
Schematic structural representation of ferrocene camphor sulphonamide complexes.

The iron-centered ferrocene camphor sulphonamide complexes (Figure 8) revealed high active anticancer agents against the aggressive breast cancer cells MDA-MB-231 [87] and several cancer lung cell lines (H1299, A549) [88] that even surpass the activity of cisplatin or tamoxifen.

#### 3.1.4. Complexes with Bidentate Camphor Ligands

Over time, many camphor and camphor sulfonic acid derivatives were synthesized. Some of them have at the substituent (R) more than one atom able to coordinate metals, thereby enabling bidentate or polydentate coordination. Chelate-type complexes can then form depending on the positions of the two atoms, the electronic characteristics, and the size of the metal core. Diamine camphor derivatives often bind metals *via* two atoms, prompting a *cis* geometry.

##### Biological Activity

**Figure 9:**
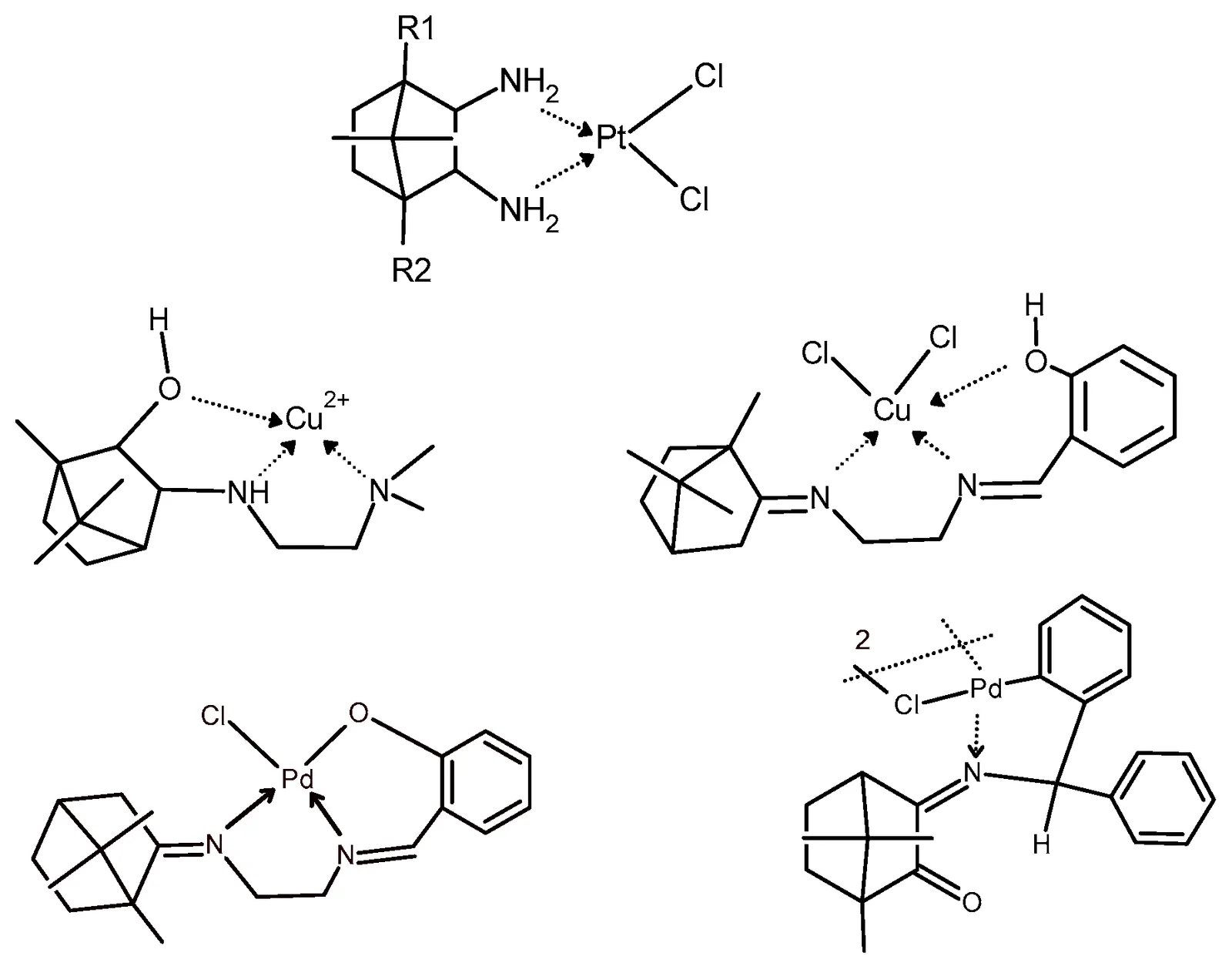
Types of camphor diamine complexes.

Among the camphor complexes that are biologically active, chirality is a parameter that highly affects their interaction with cell receptors and, consequently, their activity. Such patterns are clearly evidenced by the enantiomers of the camphor Pt(II) complexes (Figure 9 top) that show some structural analogies with cisplatin. Both the Pt(II) enantiomers are cytotoxic; however, the complexes with the ligand derived from (1*S*)-camphorquinone display much higher antiproliferative activity against HL-60 cancer cells than those derived from (1*R*)-camphorquinone [89].

The relevance of the characteristics of the metal in the biological process is once more highlighted by the fact that camphor diamine Pd(II) complexes display low or no cytotoxic activity [90] in contrast with Pt(II) complexes. However, the camphor imine Pd(II) complexes show high antifungal activity at 4 g/mL (Figure 9 bottom, left) and 0.5 μg/mL (Figure 9 bottom, right), respectively, against *Candida neoformans* (ATCC 208821) [72], consistent with higher antifungal activity than fluconazole (8 g/mL), which is one the commercially available drugs.

Similar to Pd(II), Cu(II) diamine camphor complexes (Figure 9 middle) also display antifungal activity. The activity of the Cu(II) complexes against *C. albicans* is even higher than that of amphotericin B (in use). In addition, the Cu(II) diamine camphor complexes’ antibacterial activity overcomes the resistance of *S. aureus* (MRSA) to the clinical antibiotic ciprofloxacin [91].

#### 3.1.5. Camphor Complexes with Bi-Camphor Ligands

**Figure 10:**
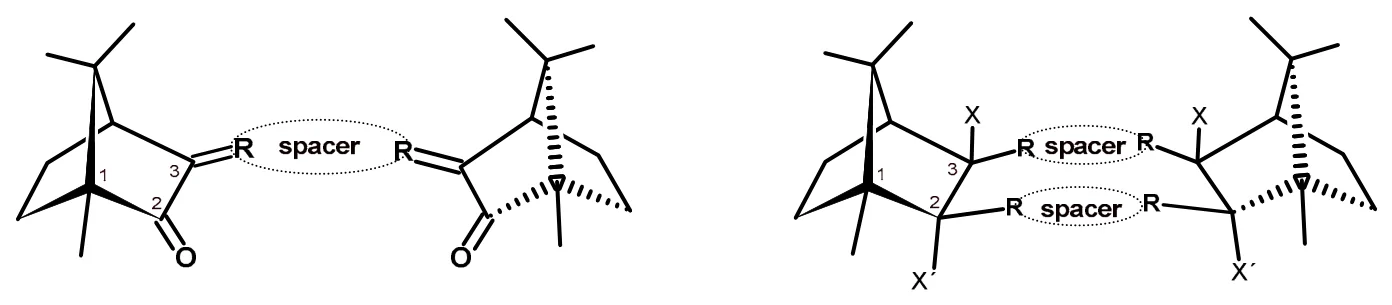
Types of bi-camphor compounds.

Bi-camphor compounds consist of two camphor units linked by a spacer (Figure 10). The linker plays a strategic role in the structural and electronic properties of the complexes [46]. Bi-camphor ligands can bind metals through one, two, or even more atoms, involving those directly attached to the camphor bicyclic skeleton and/or atoms at the spacer.

The structure of the bi-camphor ligands is particularly suitable for the formation of coordination polymers, since the bi-camphor entity can bind metals linearly, forming one-dimensional structures (1-D) or arranging as 2-D or 3-D nets. Several structural arrangements have been observed by X-ray diffraction analysis on bi-camphor complexes [92].

##### Biological Activity

Bi-camphor ligands prompt the formation of silver coordination polymers, with antimicrobial activity modulated by the characteristics of the spacer. Identical [Ag(NO_3_)(^C1^L-R-^C1^L)]) complexes display good (R = *p*-C_6_H_4_), moderate (R = *m*-C_6_H_4_), or low (R = (C_6_H_4_)_2_) activity against Gram-negative bacterial strains (*P. aeruginosa* and *E. coli*) depending on the R group (Table 4). All [Ag(NO_3_)(^C1^L-R-^C1^L] complexes display very low activity against the Gram-positive *S. aureus* strain [93]. The biological activity of bi-camphor complexes follows the trend already found for metal cores {Ag(OH)} and {Ag(NO_3_)}. The [Ag(OH)(^C1^L-R-^C1^L)] complexes perform slightly better than the [Ag(NO_3_)(^C1^L-R-^C1^L)] with identical ligands (Table 4). The Ag(I) complexes perform better towards Gram-negative than Gram-positive strains, their effectiveness varying with the spacer. At the Ag(I) complexes (Table 4), ligands that bring the two camphor units closer apparently enhance the antibacterial activity, as evidenced by the MIC values for the *E. coli* strain at the {Ag(NO_3_)} complexes, which are respectively 20 μg/mL (R = *p*-C_6_H_4_) and > 100 μg/mL R = (C_6_H_4_)_2_ (Table 4). However, not enough data exists for generalization of that assumption. Coordination polymers with suitable spacers may enhance specific interactions with DNA bases.

##### Au(I) Versus Ag(I) Camphor Imine Complexes Biologic Activity

A comparative study of the antibacterial activity of the Ag(I) and Au(I) bi-camphor imine complexes against *E. coli* strain showed that the activities of ([Au(CN)(^C1^L-R-^C1^L)]) (R = *m*-C_6_H_4_, 56.5 μg/mL) and [Ag(OH)(^C1^L-R-^C1^L)] (R = *m*-C_6_H_4_, 52.2 μg/mL) essentially do not differ. Nevertheless, against *P. aeruginosa* and *S. aureus*, the activities are largely influenced by the camphorimine ligand (Table 4). In the case of the biphenyl (R = (C_6_H_4_)_2_) bi-camphor complexes, the metal center [Ag(OH)(^C1^L-R-^C1^L)] prompts the highest activity (Table 4). Although these results are insufficient for generalization, they suggest that proper adjustment of the metal and camphor-derived ligand is necessary to optimize the antimicrobial activity [46,47].

In addition to antimicrobial activity, both Ag(I) and Au(I) bi-camphor imine complexes exhibit anticancer activity against osteosarcoma HOS (ATCC, CRL-1543) and/or melanoma A375 (CRL-1619) cell lines (Table 5).

The activity towards human dermal fibroblast HDF cells (Table 4) was also evaluated to ascertain the toxicity towards healthy cells. The IC_50_ values for complexes [Ag(NO_3_)(^C1^L-R-^C1^L)] (Table 4) essentially do not differ aside from the spacers. The cytotoxic properties are identical (R = *p*-C_6_H_4_ or R = (C_6_H_4_)_2_) and more effective than complexes based on {Ag(OH)} or {Au(CN)} central cores (Table 4).

**Figure 11:**
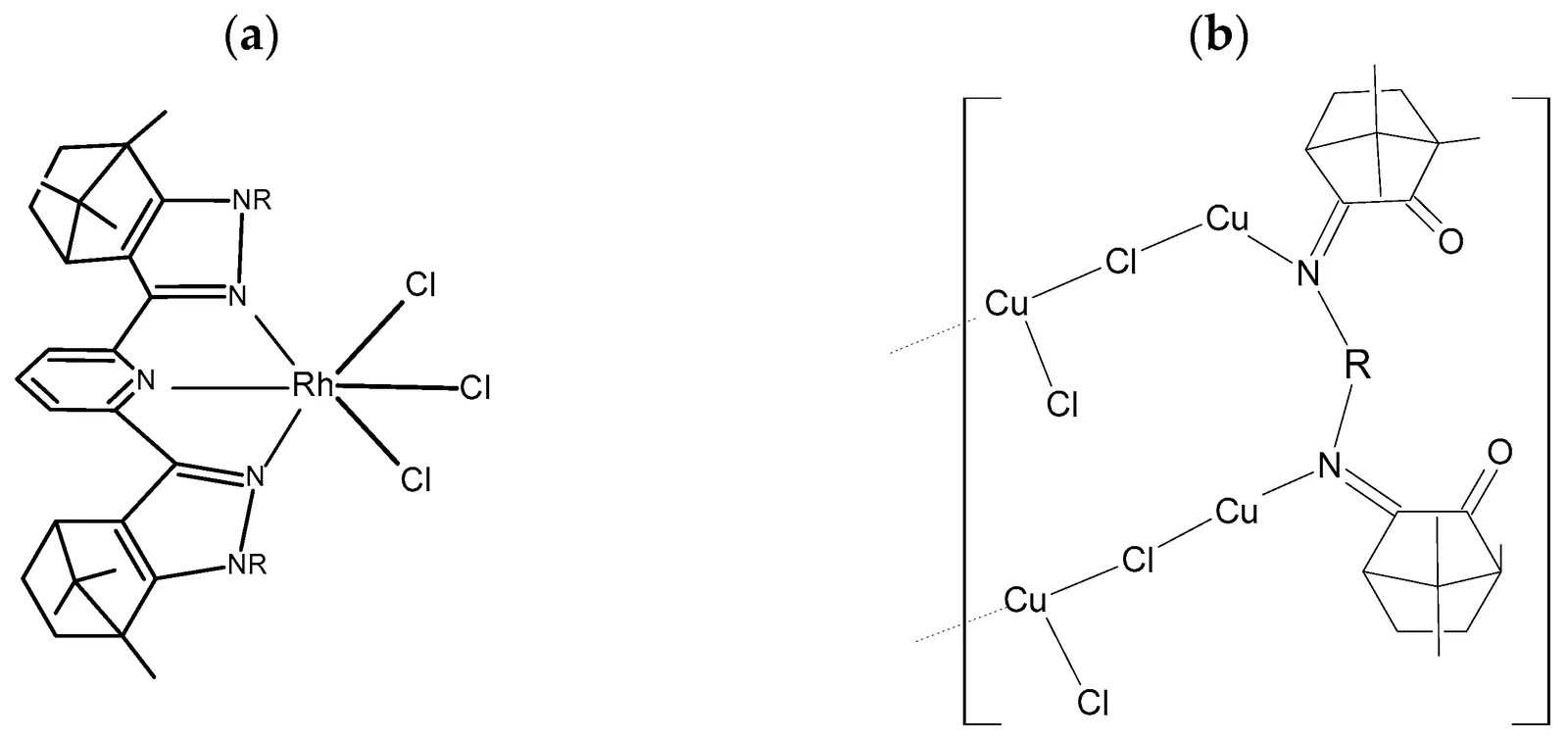
Structural arrangement of (**a**) Rh(III) and (**b**) Cu(I) camphor-derived complexes.

Apart from the above-mentioned Ag(I) and Au(I), other bi-camphor complexes reported as cytotoxically active are rhodium(III) and copper(I) complexes (Figure 11). The [Rh(R-L)Cl_3_] (R-L = bi-camphor) act against HCT-116 colorectal cancer cells with activity that depends on the R group (higher for R = Me_2_, lower for R = H_2_) at the bi-camphor bis(pyrazolylpyridine) substituent (Figure 11a) [95].

The bi-camphor imine coordination polymers [(CuCl)_4_(^C1^L-R-^C1^L)]*_n_* (Figure 11b) present moderate cytotoxic activity against colorectal cells (HT 29), highly dependent on the length of the spacer group [85], such as for the Ag(I) complexes (see above).

Not much information exists on the mechanisms that rule the cytotoxic activity of camphor-derived complexes. However, a study of the cytotoxicity of the camphor imine Ag(I) and Au(I) complexes against A2780 and OVECR3 ovarian cancer cells showed that they promote the formation of reactive oxygen species (ROS) [86], which are responsible for oxidative stress and cell apoptosis. Such a mechanism looks particularly promising to fight resistance to cisplatin that directly binds DNA.

## 4. Functionalization of Biocompatible Materials

Biocompatible materials have many applications in medicine, because they can replace, support, or repair damaged tissues and organs. Some of the applications refer to implants, tissue engineering, wound dressings, and drug delivery systems. Regarding drug delivery systems based on composites, the combination of biocompatible ceramics (e.g., hydroxyapatite, HAp) with antimicrobial and cytotoxic drugs may improve surgical outcomes by helping control infections, which are common in cancer patients due to their compromised immune systems.

Hydrogels are another type of biocompatible material whose functionalization becomes particularly attractive because they promote moist environments, absorb exudates, and support tissue regeneration. When combined with agents possessing both antimicrobial and anticancer properties, hydrogels may serve as efficient drug delivery systems for wound treatment of infections in cancer patients.

The dual antimicrobial and cytotoxic properties of some camphor imine complexes make them particularly attractive for incorporation into biocompatible materials (ceramics, hydrogels).

**Figure 12:**
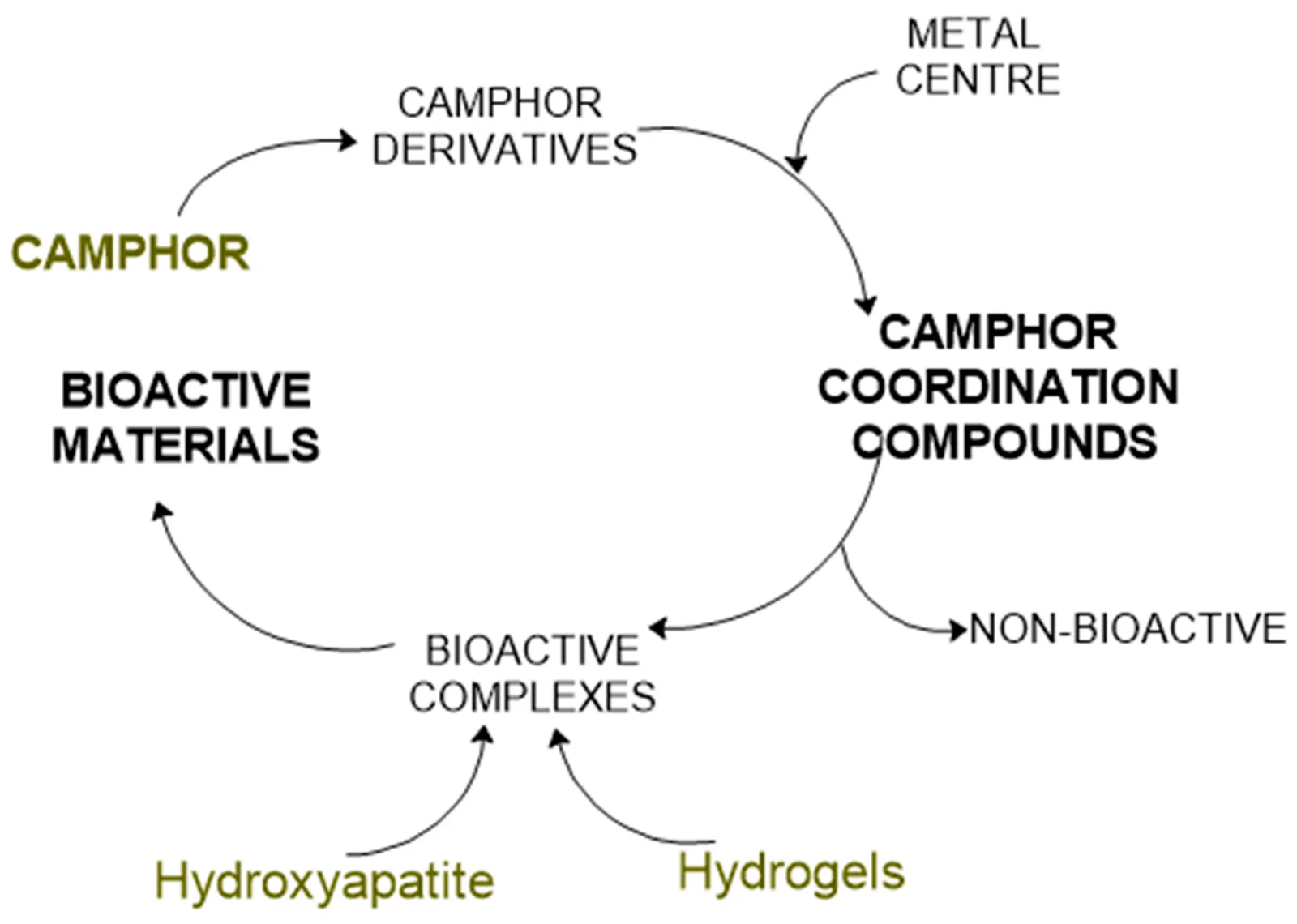
Schematic pathways from camphor to biologically active materials.

A scheme showing the pathways from camphor to biologically active materials is displayed (Figure 12).

### 4.1. Ag(I) Camphor Imine—HAp Composites

HAp is the principal constituent of bones, and thus particularly appropriate to prepare engineered materials [96] that combine antibacterial and anticancer properties aiming at bone filling of defects or upon cancer resection. An insight into the functionalization of HAp with Ag(I) camphor imine complexes ([Ag(NO_3_)L]) (L = ^C1^L-R, ^C1^L-R-^C1^L) showed that the composites functionalized by bi-camphor-based complexes keep the precursor’s anticancer activity against osteosarcoma (HOS) cells as well as the antibacterial activity against *Pseudomonas aeruginosa* and *Staphylococcus aureus* [81].

### 4.2. Ag(I) Camphor Imine Hydrogels

The absorbent and hydrophobic properties of hydrogels, together with their adjustable drug delivery characteristics, make them well suited for functionalization with antimicrobial and/or anticancer agents intended for topical applications in the treatment of skin infections and cancer (e.g., melanoma) [97,98]. Substances such as Ag(I) camphor imine complexes, which combine both antimicrobial and anticancer activities, are especially attractive for the development of wound dressings. The cytotoxicity of a few HEMA-based hydrogels against A375 cancer cells and MeWo melanoma cells and their antibacterial activity against *Escherichia coli* ATCC25922, *Pseudomonas aeruginosa* 477, *Burkholderia contaminans* IST408, and *Staphylococcus aureus* Newman showed that Ag(I)-camphor-imine-loaded hydrogels act as anticancer and antibacterial agents [83].

The results presented above demonstrate that some Ag(I) complexes exhibit both antimicrobial and anticancer activities and can be used to functionalize biocompatible materials such as hydroxyapatite and hydrogels. However, their selectivity (SI < 2) remains a significant limitation and must be improved before they can be considered suitable for therapeutic applications.

## 5. Conclusions

In the last fifty years, a wide variety of camphor-based coordination compounds were synthesized and structurally and biologically characterized. Camphor coordination compounds based on a large variety of metals have been reported. Among them, several are highly effective as antimicrobial or anticancer agents. A few combine both types of activities. The electronic and steric properties of camphor-derived ligands drive their coordination to metals and modulate their biological activities. The characteristics of the metal center play a crucial role in determining the complexes’ biological targets and, consequently, the type of biological activity they exhibit. Among the biologically active camphor-derived coordination compounds containing copper, ruthenium, palladium, and gold, the focus on Ag(I) camphorimine complexes highlighted that their antimicrobial or cytotoxic properties are fine-tuned by the type of camphorimine. Subtle modifications into the metal core of the complexes significantly influence their biological activity; for example, replacing NO_3_^−^ by OH^−^ in the coordination sphere of Ag(I) can enable activity against *Candida albicans* that was not susceptible to the corresponding nitrate-containing complexes.

The number of studies reported until now are insufficient for a systematic comparison of the antimicrobial and anticancer activities of systems based on different metals because the metal cores, the type of ligand (^Xn^L-R, *n* = 1,2 and X = A,B,C,D), the microbial strains, and the cancer cells vary considerably.

A critical parameter in the development of anticancer agents is toxicity. Effective compounds must selectively target cancer cells while minimizing damage to healthy ones. It is worth noting that some Ag(I) camphor imine compounds showed considerable selectivity.

Camphor-derived complexes can be used either directly or as components of biocompatible materials for medicinal purposes. The incorporation of Ag(I) camphorimine complexes into hydroxyapatite (HAp) or hydrogels preserve their biological activity, highlighting the potential of such materials as therapeutic anticancer or antimicrobial platforms, with certain systems demonstrating dual biological activity. These findings support further investigation of camphorimine-based matrials.

## Figures and Tables

**Table 1:** MIC values (μg/mL) of complexes [Ag(NO_3_)(^C1^L-R)_n_] ^a^ and [Ag(OH)(^C1^L-R)_n_] ^b^ (in brackets).

*R* ^c^	*E. coli* ATCC25922	*P. aeruginosa* 477	*B. contaminans* IST408	*S. aureus* Newman
NH_2_	*22*	*27*	*27*	*56*
OH	*51 (7.2)*	*52 (3.4)*	*59 (6.4)*	*>100*
C_6_H_5_	*59*	*55*	*91*	*54*
3-OH(C_6_H_4_)	*52 (12.8)*	*53 (3.9)*	*75 (8.4)*	*>100*
4-CH_3_(C_6_H_4_)	*37*	*53*	*66*	*55*
4-NH_2_(C_6_H_4_)	*40 (14.6)*	*52 (10.9)*	*>100 (16.4)*	*47*
3,5-(CH_3_)_2_(C_6_H_3_)	*65*	*121*	*>100*	*>100*

^a^ Ref [80]. ^b^ Ref [78]. ^c^ See Figure 1 for clarity.

**Table 2:** MIC values (μg/mL) for [Ag(NO_3_)(^D1^L-R)_2_] ^a^ complexes.

*R* ^b^	*E. coli*	*P. aeruginosa*	*B. contaminans*	*S. aureus*
NH_2_	*26.7*	*13.2*	*14.6*	*39.3*
C_6_H_5_	*15.5*	*6.7*	*15.1*	*41*
3-OH(C_6_H_4_)	*>100*	*>100*	*>100*	*>100*

^a^ Ref [79]. ^b^ See Figure 1 for clarity.

**Table 3:** Anticancer activity of [Ag(NO_3_)(^C1^L-R)_n_] and [Ag(NO_3_)(^D1^L-R)_2_] complexes ^a^.

					IC_50_ (μM)			
	*R*	A375	SI ^b^	A2780	SI ^b^	HOS	SI ^b^	HDF
^C1^L-R	NH_2_	*11.0 ± 3.0*	*1.7*			*5.61 ± 2.0*	*3.3*	*18.7 ± 6.3*
	OH	*17.7 ± 5.2*	*1.3*			*8.19 ± 3.0*	*2.8*	*22.7 ± 7.2*
	C_6_H_5_	*11.6 ± 2.5*	*8.6*	*3.53 ± 0.9*	*28.5*	*8.81 ± 2.5*	*11.4*	*>100*
	3-OHC_6_H_4_	*14.8 ± 3.6*	*1.2*			*12.7 ± 4.5*	*1.4*	*17.4 ± 3.6*
^D1^L-R	C_6_H_5_	*23.3 ± 7.2*	*1.4*	*0.76 ± 0.29* ^c^	*42.5*	*8.29 ± 2.0*	*3.9*	*32.3 ± 8.7*
	3-OHC_6_H_4_	*9.9 ± 4.1*	*1.6*					*15.9 ± 7.1*
	4-NH_2_C_6_H_4_	*−*	*−*			*23.7 ± 10*	*1.5*	*35.3 ± 9.1* ^d^

^a^ 24 h exposure. ^b^ SI = IC_50_(HDF cell)/IC_50_(cancer cell). ^c^ Ref. [82]. ^d^ Ref. [83].

**Table 4:** Antibacterial activity of bi-camphor (^C1^L-R-^C1^L) complexes.

	R	MIC (μg/mL) ^a^		
		*E. coli*	*P. aeruginosa*	*S. aureus*
[Ag(NO_3_)(^C1^L-R-^C1^L)]	*m*-C_6_H_4_	65	121	>100
	*p*-C_6_H_4_	20	19	73
	(C_6_H_4_)_2_	>100	86	>100
[Ag(OH)(^C1^L-R-^C1^L)]	*m*-C_6_H_4_	52.2	43	>100
	(C_6_H_4_)_2_	54	61	>100
[Au(CN)(^C1^L-R-^C1^L)]	*m*-C_6_H_4_	56.5	28.9	81.2
	(C_6_H_4_)_2_	>100	>100	>100
	Ag(NO_3_)	>100	>100	>100

^a^ Upon 24 h cell exposure.

**Table 5:** Anticancer activity of Ag(I) and Au(I) bi-camphor (^C1^L-R-^C1^L) complexes [94].

		IC_50_ (µM) ^a^		
Complex		HOS	A375	HDF
Ag(NO_3_)(^C1^L-R-^C1^L)	*p*-C_6_H_4_	3.1 ± 3.0		-
	(C_6_H_4_)_2_	3.1 ± 0.9	6.6 ± 1.6	8.5 ± 2.7
Ag(OH)(^C1^L-R-^C1^L)	*p*-C_6_H_4_	14.6 ± 4.6		41.0 ± 8.3
	(C_6_H_4_)_2_	6.7 ± 2.8		10.6 ± 3.5
Au(CN)(^C1^L-R-^C1^L)	*p*-C_6_H_4_		0.27 ± 0.04	0.65 ± 0.14
	AgNO_3_	7.2 ± 2.5		25.8 ± 5.0

^a^ Upon 24 h cell exposure.

## Data Availability

No new data were created or analyzed in this study. Data sharing is not applicable to this article.
